# Improving the reliability and availability of railway track switching by analysing historical failure data and introducing functionally redundant subsystems

**DOI:** 10.1177/0954409717727879

**Published:** 2017-09-13

**Authors:** Samuel D Bemment, Roger M Goodall, Roger Dixon, Christopher P Ward

**Affiliations:** Wolfson School of Mechanical, Electrical and Manufacturing Engineering, Loughborough University, Loughborough, UK

**Keywords:** Railways, track switch, points, reliability, availability, asset management, system failure

## Abstract

Track switches are safety critical assets that not only provide flexibility to rail networks but also present single points of failure. Switch failures within dense-traffic passenger rail systems cause a disproportionate level of delay. Subsystem redundancy is one of a number of approaches, which can be used to ensure an appropriate safety integrity and/or operational reliability level, successfully adopted by, for example, the aeronautical and nuclear industries. This paper models the adoption of a functional redundancy approach to the functional subsystems of traditional railway track switching arrangements in order to evaluate the potential increase in the reliability and availability of switches. The paper makes three main contributions. First, 2P-Weibull failure distributions for each functional subsystem of each common category of points operating equipment are established using a timeline and iterative maximum likelihood estimation approach, based on almost 40,000 sampled failure events over 74,800 years of continuous operation. Second, these results are used as baselines in a reliability block diagram approach to model engineering fault tolerance, through subsystem redundancy, into existing switching systems. Third, the reliability block diagrams are used with a Monte-Carlo simulation approach in order to model the availability of redundantly engineered track switches over expected asset lifetimes. Results show a significant improvement in the reliability and availability of switches; unscheduled downtime reduces by an order of magnitude across all powered switch types, whilst significant increases in the whole-system reliability are demonstrated. Hence, switch designs utilising a functional redundancy approach are well worth further investigation. However, it is also established that as equipment failures are engineered out, switch reliability/availability can be seen to plateau as the dominant contributor to unreliability becomes human error.

## Introduction

This paper demonstrates the possible reliability benefits from the adoption of functionally redundant subsystems in railway track switching, using baseline data from a modern, high-performance rail network. A background in the existing track switch design and practice is first established. The reliability performance of existing installations is examined by using a dataset provided by the UK infrastructure owner, Network Rail. These data are analysed to provide failure distributions of switch installations, and individual subsystems thereof, in the section titled ‘Establishing Failure Rates and Distributions’. An RBD (reliability block diagram) modelling approach is used to establish the analytical reliability (static) and availability (dynamic) benefit of applying a multi-channel architecture to track switch designs, to provide a degree of redundancy. The results are presented and examined in the ‘Analysis’ section and show that the approach can deliver track switching with operational reliability much enhanced when compared to existing installations.

### Background

Rail networks requiring more than a single vehicle upon a single line are dependent upon the ability to provide multiple routes for traffic. Switches (UK: Points) serve this purpose, allowing the track to merge and diverge. The standard switch design, in use throughout the world, consists of two ‘switch blades’ upon a suitable supporting structure, which are able to slide laterally between two ‘stock rails’. Whilst recognising that switch actuation has evolved over time – from mechanical rods and levers to more modern electro-mechanical or electro-hydraulic designs – the basic mechanical arrangement of switches has remained identical since the first railways were envisioned. An extensive description of switch design is provided by Morgan.^1^

Despite their necessity, switch failures can rapidly cripple rail operations. Unlike road transportation, where vehicles can simply steer around failed vehicles or roadway, in a guided transport system the vehicles are reliant upon switches in order to change direction. This means that a switch failure renders all vehicles upon direct approach unable to move until it is repaired. This disruption is magnified where no wider diversionary route is available, and the consequent ‘knock-on delays’ increase rapidly. Ison et al.^2^ list some UK routes now running at over 90% capacity, and similar situations exist upon major commuter railways in continental Europe. In such situations, the effects of switch failures are profound. Literature explores optimisation options for managing perturbed traffic to reduce these knock-on delays, for instance the work of Pellegrini et al.^3^ Eliminating the cause of delays and perturbations by preventing switch failures is another approach explored in literature, for instance by García et al.,^4^ and Silmon and Roberts^5^ – both papers exploring condition monitoring algorithms and architectures with the goal of reducing failures. García et al.^6^ also explore a move to reliability-centred maintenance, rather than the periodic maintenance regime currently in place. However, these approaches do not render the system truly ‘fault tolerant’ and are instead aimed at reducing the incidence of failure through predicting when failures are likely to occur. In addition, with a single-point-of-failure system and limited time/budget to cope with false positives, these strategies may have a diminishing return when looking to enhance system availability, a problem which is discussed by Bemment et al.^7^

### Fault tolerance

A fault tolerant system is able to prevent faults developing into failures through design, as described by Blanke and Schröder.^8^ This design can include:

- Systems which isolate or compensate for faulty components
- Functional design providing a level of capability without given components
- Parallel channels which can each perform a given set of requirements alone

In most cases, the first two options cost less in monetary terms, but some safety critical systems are forced to follow the third principle, despite cost/weight penalties, to achieve the level of reliability/integrity deemed necessary for the safe operation of the system. Fault tolerance is important in safety-critical engineering, such as in aircraft, bridges, cars and nuclear power. Without fault tolerance, many designs could not function to the standard required by their regulatory environment. A prime example is aircraft flight control surfaces, which would typically have triplex or quadruplex sensor, control and actuation systems to ensure control of the aircraft in the case of concurrent failure of several actuation systems. Literature explores options for fault tolerance at rail junctions, for instance by Ursani et al.^9^ However, this approach is related to tolerance of faults in the optimum scheduling of traffic by reconfiguring the signalling, and not the tolerance of asset failures.

Other applications involving safety-critical systems have utilised redundancy as a method of achieving high-availability and/or fault-tolerant operation, as described in Hecht^10^ and Isermann.^11^ Redundant systems have seen use in the rail sphere, a successful and internationally adopted example being the architecture of solid state interlocking.^12^ This has provision for both fault detection and tolerance; triplex individual processing units vote and any singular disagreement in output is discarded, with the whole system continuing to function at a degraded level. This approach has not yet, however, been adopted for physical elements of the track switching system.

## Current practice

### Physical arrangement

**Figure 1.**
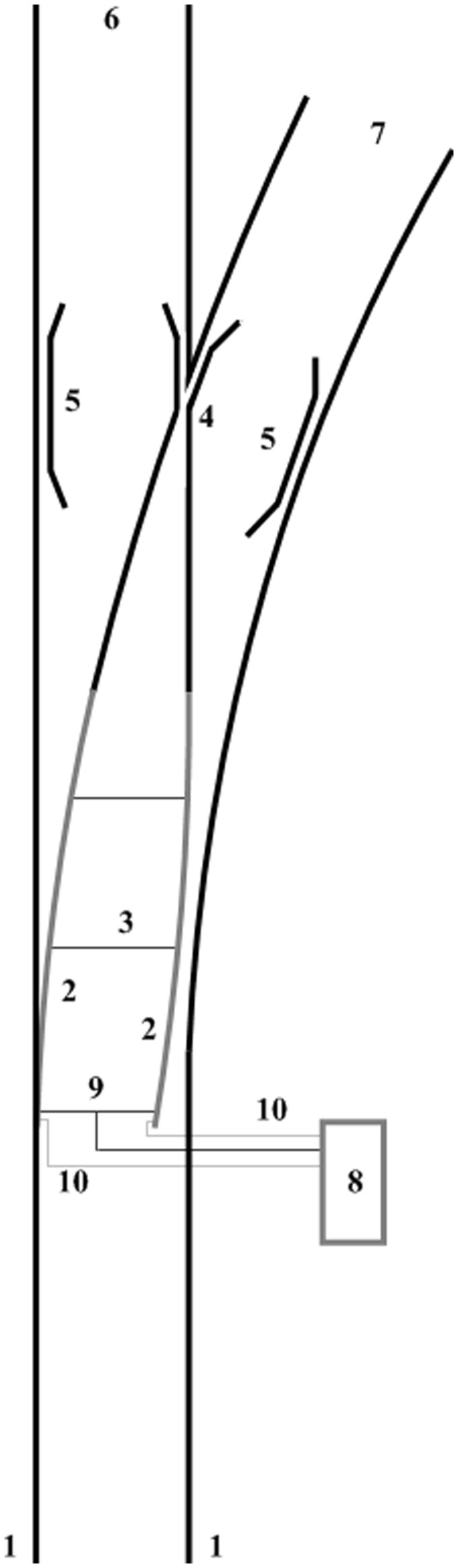
Typical switch arrangement, taken from Bemment et al.^13^ 1: stock rails; 2: moveable switch rails; 3: stretcher bars; 4: common crossing; 5: check rails; 6: straight route; 7: turnout route; 8: POE (points operating equipment), line-side type shown; 9: drive bar and drive stretcher; 10: detector rods.

Figure 1 shows the diagram of a typical UK installation, consisting of two stock rails, two switch rails and a common crossing, fastened by clips, bolts and/or chairs to supporting bearers of wood or concrete, themselves supported upon a bed of ballast or concrete slab. The stock rails are securely fixed to prevent movement, whilst the ends of the switch rails are free to slide upon supporting cast iron chairs, their movement restricted by the attached stretcher bars and the lock and drive arrangement provided by the POE (points operating equipment).

There are several different designs of POE (see ‘Subsystem Identification’ section) which are located variously in between the running rails, at the line side or a combination of both. Detection rods and/or switches provide feedback that the blades have reached an acceptable position (and are locked) to the POE, and subsequently the interlocking system.

Higher line speeds necessitate shallower divergence angles due to limitations on lateral acceleration and cant deficiency at the common crossing. This in turn requires longer switches. Longer designs require multiple actuation points upon the switch blades to ensure the entire moveable blade length (up to around 40 m in some designs) is positioned correctly for the passage of traffic. This actuation is provided either by a power take-off from the main actuator or additional actuators situated along the length of the movable portion – though crucially, not in a redundant configuration because all actuators must be operating correctly.

The principles of power point operation were established in the early 20th century as the power point machines and electric signalling became widespread. The operating principles are extensively described by Hadaway.^14^ The principles have more recently been combined into an industry standard, in *GKRT0062*.^15^ For the UK case, the turnout is commanded to be in either of two positions – labelled ‘normal’ or ‘reverse’ – at all times by the interlocking. If the required position changes, the command signal from the interlocking will change over, triggering a sequence of events in the line-side control circuitry and POE which is referred to as the ‘move-lock-detect’ cycle, which occurs as follows:

1. Detection of the current position is broken, allowing the actuator to move.
2. The actuator begins movement, first unlocking the switch blades, allowing them to move freely.
3. The actuator moves both switch blades simultaneously to their commanded position.
4. The blades reach their commanded position, and the actuator ceases to move them.
5. The actuator re-engages the locking mechanism.
6. Detection is made for both switch blades and the lock, automatically shutting down and isolating the actuator.

Most POE designs offer combined actuation, locking of both switch rails and full detection through single combined motion mechanisms. The turnout is considered unsafe without a detected position, even though both switch rails may be locked in the correct position. Without detection, the interlocking cannot clear the route, and trains are prevented from passing the switch. This has the effect that, even for functional switches, signals on the approach must show restrictive aspects when the switch is moving, representing a capacity constraint explored by Bemment et al.^16^ and in a report by the Transportation Research Board.^17^

It is beyond the scope of this paper to provide a detailed discussion of switch design and operation; this is extensively covered in literature. Full details of switch design and operation are presented by Morgan^1^ and Cope and Ellis.^18^ Bemment et al.^7^ provide a list of the functional requirements of track switching solutions.

### Asset reliability: The magnitude of the problem

Data are published by the United Kingdom's ORR (Office of Road and Rail^19^) pertaining to the reliability of the existing switch installations. An excerpt of these data is reproduced in Table 1 to illustrate the magnitude of the issue of switch reliability facing the GB mainline. This table includes a breakdown of the number of failure incidents over financial years (FY) 07/08–11/12. The delay minute total is the sum of all delays, to all trains, caused as a direct result of an asset failure. The cost data are calculated as the sum of the total of delay minute compensation, essentially the compensation paid by the network custodian to the train operators for unscheduled downtime. This figure does not allow for the subsequent economic impact of any such failure. It can be observed that track switch failures are the second biggest contributor – both financially and in time – after track faults, at around £26m/FY.

**Table 1.** Cost and delay minute incursion for various asset types.

	Cost		Delay minutes	
Asset type	(MGBP)	%	(1,000 s)	%
Track	131.9	19.2	3,977	18.8
Switches	121.1	17.6	3,874	18.3
Track circuits	99.5	14.5	3,208	15.2
Signalling system	95.2	13.9	2,727	12.9
Electrification	75.4	11.0	1,529	7.2
Signals	40.2	5.9	1,428	6.8
Cabling	37.4	5.4	1,013	4.8
Track TSRs	34.5	5.0	1,630	7.7
Axle counters	18.5	2.7	495	2.3
Level crossings	13.2	1.9	521	2.5
Other signalling	11.6	1.7	363	1.7
Telecoms	9.1	1.3	363	1.7
Totals	687.8		21,128	

Values are totals for period FY07–08 and FY11–12. Public domain obtained from Office of Road and Rail.^16^ MGBP: Great Britain Pounds; TSR: Temporary Speed Restriction.

## Baseline data

### Mainline failure logging

Network Rail keeps records of all known asset failure events in a database called ‘FMS’ (Fault Management System). This database contains many fields which are relevant to this study. The database records both faults and failures, identifying the difference between the two with a ‘criticality index’ between 1 and 4. Criticality indices 1 to 3 represent failures requiring immediate rectification. Index 4 is a known fault, which will need rectifying when possible, but one which has not yet developed to a system failure. The data held by FMS do not include the number of delay minutes incurred (or subsequent monetary cost) for individual failure events; these data are held in a separate database called TRUST, without historical cross-referencing. Data are entered by human operators, often line-side and in difficult conditions, and as such there is a significant portion of records which may be incomplete or considered corrupt. Data accuracy improves considerably after 2009 when free-text entry was replaced by option selection in several fields.

### Dataset for this study

For this study, Network Rail provided a dataset extracted directly from FMS. This consisted of a database query for all entries pertaining to Points for dates between 1 April 2008 and 17 September 2011. This resulted in 39,339 fault/failure records, which were supplied in CSV format. The population of switches on the UK mainline was 21,602 in 2011,^20^ but has stayed broadly constant during the period, and populations will be considered constant throughout this analysis. These data correspond to a cumulative operating time of 74,800 years.

### Cleansing the dataset

Since the data were directly extracted from the database, extensive processing was required before use. Of the obtained fields, several fields contain duplicate information, but not every field was populated for every record; therefore, identifying these duplicates was important for data cleansing. First, a script was created which back-populated missing fields based on the contents of populated entries, in order to give a more complete dataset. Certain switch types were then excluded from the data due to specialist applications, for example, those with very small populations or obsolete technology already being phased out (e.g. pneumatic machines). Hydraulic derailers, identified as switches in the database, were also discounted. Table 2 shows the number of records discounted for each reason.

**Table 2.** General statistics showing size of pre- and post-cleansing dataset obtained from Network Rail for the period 1 April 2008 and 17 September 2011.

Total records obtained/analysed	39,339
Minus	
Blank/insufficient data/corrupted/irrelevant	966
Pneumatic machines	1,519
GRS Type 5 machines	253
Remaining useable data records	36,601
Of which:	
Criticality 1–3 (service affecting failure)	17,603
Criticality 4 (non-service affecting)	18,998
Within the useable data	
Unique Switch assets identified	12,042
(from an analysed population of)	19,915
Switches without a failure event in the period	9,560
Showing only a single failure event	4,756
Showing two failure events	2,516
Showing three failure events	1,567
Showing four or more failure events	3,203

### Subsystem identification and event assignment

**Figure 2.**
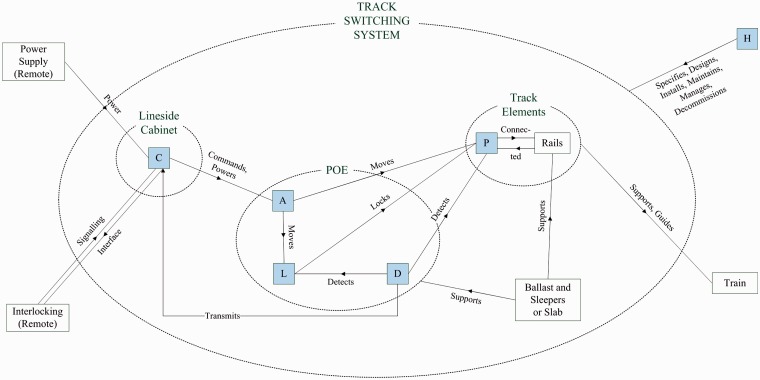
System context diagram showing relationship between functional subsystems, and where appropriate, their relationship with the wider railway environment. POE: points operating equipment.

Understanding the design and operation of switches allows their decomposition into a number of functional subsystems for further analysis. The division of functionality into subsystems is in some cases, however, an exercise of engineering judgement, as some components in switch designs can be seen to cross the established subsystem boundaries. The subsystem divisions used for the modelling presented herein were established as part of a series of workshops held in 2011–2013, with representatives from across the GB rail industry, detailed in Bemment et al.^7^ The following functional subsystems are identified; a shorthand identifying letter is adopted for each, and the relationship between these subsystems is shown in Figure 2:

- (A) *Actuation*: Elements for moving the track between positions and actuating the locking mechanism: actuator/gearing, transfer of power/motion, including backdrive arrangements.
- (C) *Control/Power*: Elements which locally control the other subsystems and provide power: signalling relays, transformers, back-up supplies.
- (D) *Detection*: Elements which sense and transmit the position of the switch rails and lock back to the control system: microswitches, contacts, Linear Variable Differential Transformer(LVDT).
- (H) *Human*: Humans responsible for the design, maintenance and operation of the switch, including fault finding and repairs.
- (L) *Locking*: Elements which prevent the un-commanded movement of one or both switch blades: lock bodies, lock dogs, associated mechanisms.
- (P) *Permanent Way*: Elements which support and guide vehicles, maintain the gauge and alignment of the track: stretcher bars, track clips, slide chairs.

Several designs of POE are analysed. ‘Mechanical’ refers to those switches driven by rod from signalbox levers or ground frame, and subsystem interactions therefore differ slightly from Figure 2. HW and W63 designs are both electromechanical in nature. They are from different suppliers and have different internal designs and components. The source data do not explicitly distinguish between them, thus they remain grouped herein. For the same reason, Clamplock designs are grouped with Hydrive designs. Both Clamplock and Hydrive are hydraulic POE designs with a separate power pack and hoses linked to rams between the running rails. The HPSS (high-performance switch system) machines are the newest design on Network Rail infrastructure and use a screw jack actuator.

By comparing the switch type, assembly type and component type identified in each dataset record, each failure event can be assigned to a particular subsystem, for a given POE type. The total number of records in each assignment is shown in Table 3. Note that it is not possible to separate the Locking and Actuation functions in the HPSS machine, as the locking is carried out by the same screw jack mechanism within the actuation element; all failures have thus been grouped under the Actuation category. Failure counts for ‘Control/Power’ upon mechanical switches are unrealistically low. This does not indicate a much higher reliability, but instead that not every mechanical switch is fitted with electronic interlocking; at the time of analysis, data were not available on the portion of the population with/without this feature.

**Table 3.** Switch populations and fault/failure incidence count for each subsystem classification within each switch type, for the period 1 April 2008 and 17 September 2011.

POE type	(Pop)	All recorded fault/failure incidents (FRI)							Service affecting failures only (SAF)						
		A	C	D	H	L	P	Total	A	C	D	H	L	P	Total
Clamplock/ Hydrive	6,852	5,412	1,780	2,358	256	5,129	885	15,821	2,494	921	1,120	115	2,235	346	7,231
HPSS	599	345	548	872	32	–	20	1,817	175	328	601	17	–	13	1,134
HW/W63	9,153	5,799	2,607	2,681	349	1,483	1,687	14,606	2,874	1,466	1,327	178	778	655	7,278
Mechanical	3,311	2,102	52	1,251	50	837	66	4,357	656	23	494	21	320	18	1,533
Total	19,915	13,658	4,987	7,162	687	7,449	2,658	36,601	6,200	2,738	3,542	331	3,332	1,033	17,176

POE: points operating equipment.

## Establishing failure rates and distributions

### Operational reliability – Definition

It is necessary to distinguish between *unsafe* failures (i.e. resulting in a system in an unsafe state), *operational* failures and *faults* when discussing the reliability of safety critical systems. Literature on the topic can cause confusion by representing any and all by the terms Mean Time Between/To Failure, abbreviated ‘MTBF’ or ‘MTTF’. The time between unsafe failures is not considered any further herein, but these are essentially undetected failures which make the switch dangerous to traffic. These would be included in operational failures, but are comparatively so rare as not to affect the analysis.

- *MTTSAF* – Mean Time to Service Affecting Failure, describes how often the system can be expected to suffer a failure which is service affecting (operational reliability).
- *MTTFRI* – Mean Time to Fault Requiring Intervention, describes the frequency that maintenance crews must visit the asset to rectify faults *and* failures.
- *MTTR* – Mean Time to Repair – the mean time from notification of a failed asset (or subsystem thereof) to returning that asset or subsystem to an as-good-as-new state.

MTTSAF and MTTFRI figures are included here as they are used as a de-facto measure within industry; however, when comparing skewed distributions, the 50% survivor function, or *B*_50_, provides a better indicator. Unless otherwise stated, the *B*_50_ refers to service affecting failures. *B*_50_ indicates the time at which half the population is expected to have failed, i.e. the median.

MTTR is difficult to quantify as the actual repair time for operational failures (i.e. the time the switch is unavailable following a failure in use) is not recorded by the infrastructure operator. For this modelling exercise, the mean number of ‘delay minutes’ per incident will be used – 106 min. The calculation, and attribution, of delay minutes to particular faults is not through a particular scientific process, but values provided in Table 1 are used herein to provide a first estimate of MTTR of the correct order of magnitude. More accurate knowledge of the distribution of MTTR figures would be of significant benefit to such a study, especially in the case of different subsystems having very different repair times. However, with the absence of further information, the influence of this figure upon the results has been mitigated by assuming a constant throughout.

### Constant failure rates

Assuming a constant failure rate, the well-known equations (equations (1) to (3)) presented by Hecht^10^ can be used to calculate MTTSAF and MTTFRI figures for each subsystem and assembly using the data in Table 3. Equation (1) expresses the sum of the operational time between events (TTF) and observational suspensions (*TTS*) for each failure event (*NFT*) in the total (*N*_*SAF*_ or *N*_*FRI*_) and observational suspension event (*NST*), divided by the number of observed failure events (*N*_*F*_). An observational suspension, sometimes referred to as a censored lifetime, is a subsystem reaching the end of the observation window in a functional or repaired state; the asset is known not to have failed in that period, but its exact point of failure subsequent to the observation period is unknown. In the case of a fixed observation window across all assets, as here, this can be simplified to equations (2) and (3), including the known population (*P*) and observation time window (*T*). For a constant failure rate, the rate can be expressed as the reciprocal of the mean, as per equations (4) and (5).

(1) $$MTTF=\frac{\sum_{i=1}^{NFT}TTF_{i}+\sum_{j=1}^{NST}TTS_{j}}{N_{F}}$$

(2) $$\mathrm{MTTSAF}=\frac{P\times T}{N_{\mathrm{SAF}}}$$

(3) $$\mathrm{MTTFRI}=\frac{P\times T}{N_{\mathrm{FRI}}}$$

(4) $$\lambda_{\mathrm{SAF}}=\frac{1}{\mathrm{MTTSAF}}$$

(5) $$\lambda_{\mathrm{FRI}}=\frac{1}{\mathrm{MTTFRI}}$$

(6) $$\mathrm{SAF}B_{50}=\frac{\ln2}{\lambda_{\mathrm{SAF}}}$$

(7) $$\mathrm{FRI}B_{50}=\frac{\ln2}{\lambda_{\mathrm{FRI}}}$$

The results of these calculations are tabulated in Table 4. Mean times calculated in this way are indicative only of the relative unreliability contribution of each subsystem to the whole system and of the relative reliability of the different POE designs. To provide baseline values for comparison with variable-frequency analysis later in the paper, the *B*_50_ values of the same assets are shown in Table 5. The *B*_50_ values in Table 5 have been derived from equations (6) and (7), which are valid under the assumption of constant failure rates only. All *B*_50_ values calculated as part of the later, variable failure rate analysis are established as part of the Monte-Carlo modelling process.

**Table 4.** MTTFRI and MTTSAF figures for functional subsystems of different POE types upon the GB mainline network, calculated using data sampled between 1 April 2008 and 17 September 2011.

	MTTFRI (years)							MTTSAF (years)						
	A	C	D	H	L	P	All	A	C	D	H	L	P	All
Clamplock/Hydrive	4.4	13.3	10.1	92.7	4.6	26.8	1.5	9.5	25.8	21.2	206.1	10.6	68.5	3.3
HPSS	6.0	3.8	2.4	65.0	n/a	102.2	1.1	11.8	6.3	3.5	121.1	n/a	157.4	1.8
HW/W63	5.5	12.1	11.8	90.6	21.4	18.8	2.2	11.0	21.6	23.9	178.4	40.7	48.3	4.4
Mechanical	5.5	219.9	9.2	230.9	13.7	174.3	2.6	17.5	490.6	23.2	548.3	35.8	621.4	7.5
All	5.0	13.8	9.6	100.3	9.3	25.9	1.9	11.1	25.2	19.5	208.4	20.7	66.7	4.0

*MTTSAF*: mean time to service affecting failure, describes how often the system can be expected to suffer a failure which is service affecting (operational reliability); *MTTFRI*: mean time to fault requiring intervention, describes the frequency that maintenance crews must visit the asset to rectify faults *and* failures.

**Table 5.** B50 figures corresponding to the MTTSAF and MTTFRI figures presented in Table 4.

	FRIB50 (years)							SAFB50 (years)						
	A	C	D	H	L	P	All	A	C	D	H	L	P	All
Clamplock/Hydrive	3.0	9.2	7.0	64.3	3.2	18.6	1.0	6.6	17.9	14.7	142.8	7.4	47.4	2.3
HPSS	4.2	2.6	1.6	45.1	n/a	70.8	0.8	8.2	4.4	2.4	83.9	n/a	109.1	1.3
HW/W63	3.8	8.4	8.2	62.8	14.8	13.0	1.5	7.6	15.0	16.5	123.6	28.2	33.5	3.0
Mechanical	3.8	152.4	6.4	160.0	9.5	120.8	1.8	12.1	340.0	16.1	380.0	24.8	430.7	5.2
All	3.5	9.6	6.7	69.6	6.4	18.0	1.3	7.7	17.4	13.5	144.5	14.3	46.2	2.8

### Lifetime distribution selection

A range of suitable variable failure rate models were evaluated upon the data, including 2P- and 3P-Weibull, Gamma, Normal and 1P- and 2P-Exponential, using a correlation coefficient test and the maximum likelihood estimation (MLE) approach described below. For each subset of data, the 2P- or 3P-Weibull distribution proved the best fit for the data.

The Weibull distribution is a general purpose reliability distribution used to model times-to-failure of electronic and mechanical components, equipment or systems. The 2P-Weibull distribution, described by Hecht,^10^ has two parameters, the shape factor *β* and the characteristic life, or scale parameter, *η*. Equations (8) and (9) show the relationship between the failure frequency and failure rate and the distribution parameters at given time, *t. β* indicates whether a subsystem has a tendency towards early-life, ‘infant mortality’ failures (β<1), constant failure rate (*β* = 1) or late-life, ‘wear-out’ failures β>1. *η* indicates the scale of the probability density function in time, a larger *η* indicating a longer time to failure; though noting that *η* values are not directly comparable, as they depend upon the corresponding *β* value. The 3P-Weibull distribution also requires *γ*, which represents an offset in time for the origin of the curve.

In the analysed cases where the 3P-Weibull distribution proved most suitable, it did so with an offset parameter which was insignificantly small; therefore, the 2P-Weibull was selected as the most suitable distribution for this modelling exercise. Published work by Rama and Andrews^21^ obtains a similar though more targeted dataset from the same source and fits distributions to the grouped data. The work also establishes that the Weibull distribution is the most appropriate distribution to model switch component lifetimes and also selects the two-parameter model over the three-parameter model for the same reasons.

One drawback of the Weibull function is that it is not capable of exhibiting non-monotonic shapes in the hazard function. This means the bathtub curve, typically observed over a whole component and population lifetime, cannot be replicated. However, this drawback is offset by the sample period being across a range of component ages, and the use of confidence intervals to give an indication of the goodness-of-fit of the distributions identified.

Rama and Andrews^21^ also list a number of assumptions which need to be made when modelling lifetime distributions in this way, namely:

1. Each failure is rectified by repairing or replacing the failed component.
2. Equipment can either be in a good (operational) or bad (failed) state.
3. Repair/replacement returns components to the as-good-as-new state.
4. Times to failure of individual components are independent of each other.
5. Time duration of the component in the failed state is insignificant in comparison to the functioning period.
  (8) $$f(t)=\frac{\beta }{\eta }(\frac{t}{\eta }{)}^{\beta -1}e^{-(\frac{t}{\eta })\beta }$$
  (9) $$\lambda (t)=\frac{\beta }{\eta }(\frac{t}{\eta }{)}^{\beta -1}$$
  (10) $$B_{50}=\eta (\ln(2){)}^{\frac{1}{\beta }}$$
  (11) $$\mathrm{MTTSA}F_{\mathrm{Weibull}}=\eta_{\mathrm{SAF}}\Gamma (1+(\frac{1}{\beta_{\mathrm{SAF}}}))$$

### Distribution fitting process

**Figure 3.**
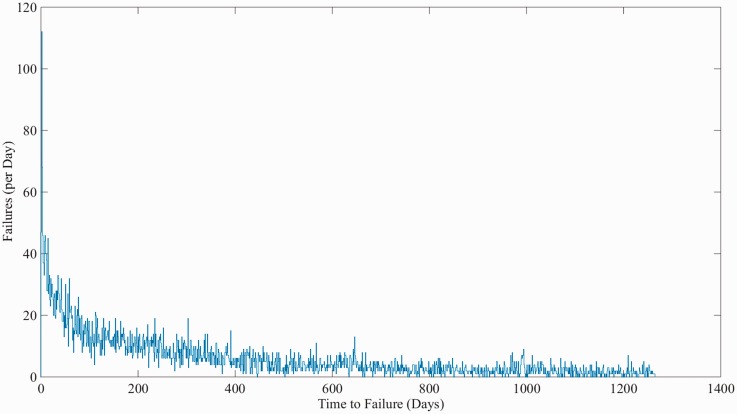
Histogram of all Clamplock/Hydrive failure intervals.

**Figure 4.**
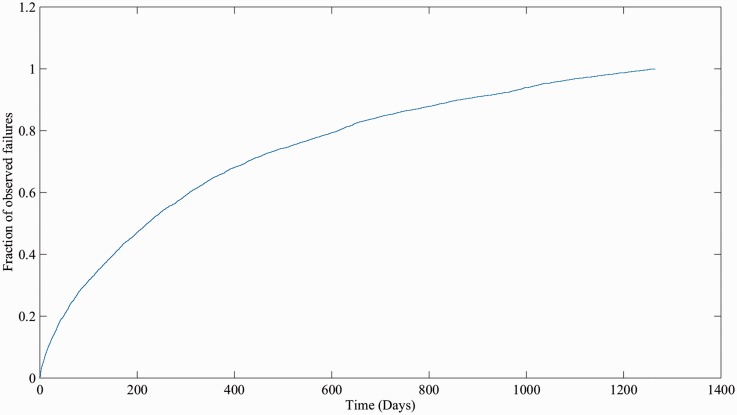
Cumulative portion of all Clamplock/Hydrive failure events over observed time.

First, records were grouped by each unique asset and then placed upon failure event timelines. The output from this process is, for each established subsystem/switch type group, an array of ‘time to event’ figures, where the event is either a failure or suspension of test. This process was automated using an iterative script; however, due to historical changes in data entry methods, significant manual intervention was also required. Figure 3 shows a histogram of the time-to-failure data for all Clamplock/Hydrive failures. Figure 4 shows the cumulative proportion of observed failures over time; as the gradient of the plot is shallower with time, it indicates that the failure pattern tends towards infant mortality.

**Figure 5.**
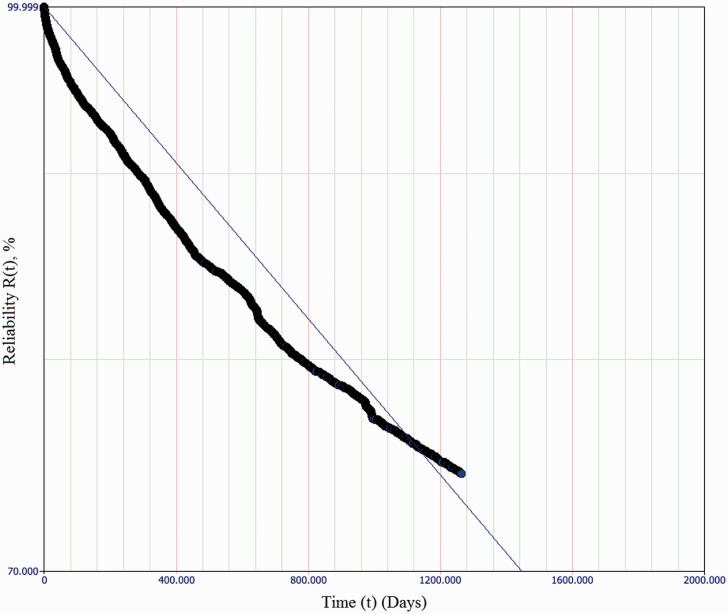
Best-fit line for exponential failure distribution (i.e. constant failure rate) of Actuation subsystem of HW/W63 machine class, showing a considerable deviation from observed data.

**Figure 6.**
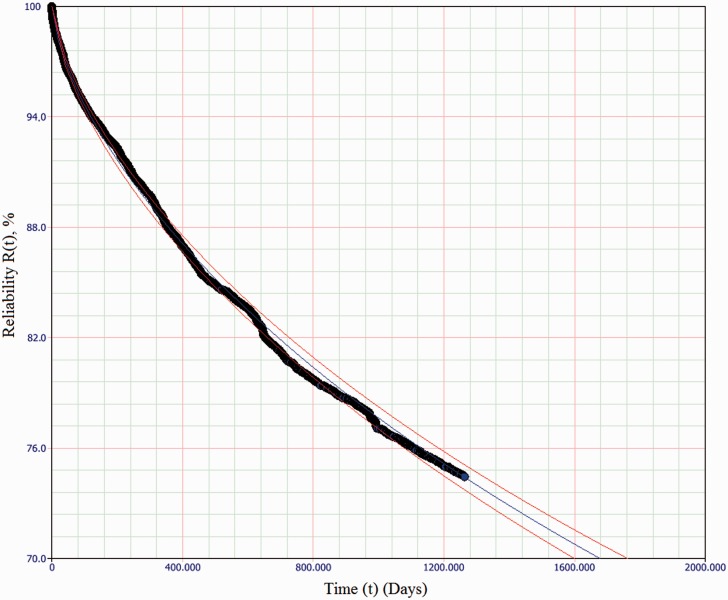
2-P Weibull failure distribution (β=0.662,η=7953) and 90% confidence interval of Actuation subsystem of HW/W63 machine class, showing a much closer correlation to the observed data than Figure 5.

The output arrays can be used as the input to an MLE algorithm. MLE is an estimator technique suitable for data that have a relatively high portion of observational suspensions; the proportion of observational suspensions in these data prevents the use of other techniques, e.g. rank regression. MLE works by developing a likelihood function based on sampling the data and by finding the values of parameter estimates that maximise this likelihood function. It is an iterative method. The process is well established and documented, for example by Scholz.^22^ *β* and *η* values were established (for service affecting failures only) in each of the subsystems in each switch classification, and the computed values are tabulated in Table 6. Values of the parameters at the extremes of a 90% confidence interval are also provided to indicate the goodness of fit. Table 6 also lists the computed *B*_50_ values for each subsystem, and (for the sake of compatibility with existing practice only) the computed MTTSAF values, also with 90% confidence intervals. The calculation of these values for a given 2P-Weibull distribution uses equations (10) and (11), where Γ represents the Gamma function. An example of a fitted exponential model for failure distributions, for the Actuation subsystem of an HW/W63 machine type, is plotted in Figure 5. Figure 6 is a plot of the same failure data, with a fitted 2P-Weibull distribution. These two plots illustrate the relative unsuitability of the constant failure rate model with these data.

**Table 6.** Calculated values of *β, η*, B50 and MTTSAF, including 90% confidence intervals, tabulated by POE type and subsystem type.

		Service affecting failures only						
		A	C	D	H	L	P	All
Clamplock/Hydrive	*β*_lower_	0.716	0.750	0.674	1.268	0.709	0.809	0.636
	*β*	0.738	0.789	0.707	1.477	0.732	0.882	0.647
	*β*_upper_	0.760	0.829	0.740	1.707	0.755	0.959	0.658
	*η*_lower_	4,639	12,822	13,920	13,267	5,329	26,688	1,332
	*η* (days)	4,940	14,739	15,941	19,418	5,713	35,548	1,374
	*η*_upper_	5,276	17,104	18,434	30,914	6,144	49,022	1,417
	$B_{50,\mathrm{lower}}$	7.8	22.5	23.2	29.3	8.9	49.8	2.1
	B_50_ (years)	8.2	25.4	26.0	41.5	9.5	64.3	2.1
	$B_{50,\mathrm{upper}}$	8.7	28.8	29.4	63.5	10.1	85.6	2.2
	MTTSAF_lower_	15.3	40.0	47.4	33.3	17.7	77.5	5.0
	MTTSAF (years)	16.3	46.2	54.8	48.1	19.0	103.7	5.2
	MTTSAF_upper_	17.5	54.0	64.0	75.4	20.6	143.7	5.3
HPSS	*β*_lower_	0.594	0.519	0.599	0.641	n/a	0.809	0.545
	*β*	0.671	0.564	0.637	0.967	n/a	1.253	0.569
	*β*_upper_	0.754	0.612	0.676	1.389	n/a	1.836	0.593
	*η*_lower_	6,481	3,496	1,492	15,058	n/a	8,756	644
	*η* (days)	8,641	4,243	1,671	47,391	n/a	22,730	701
	*η*_upper_	12,130	5,267	1,887	326,420	n/a	119,097	766
	$B_{50,\mathrm{lower}}$	22.9	5.1	2.3	31.5	n/a	19.6	0.9
	B_50_ (years)	31.2	6.1	2.6	88.9	n/a	46.5	1.0
	$B_{50,\mathrm{upper}}$	45.2	7.3	2.9	509.1	n/a	208.7	1.1
	MTTSAF_lower_	10.8	15.3	5.7	41.7	n/a	22.8	2.8
	MTTSAF (years)	13.7	19.1	6.4	131.7	n/a	58.0	3.1
	MTTSAF_upper_	18.2	24.4	7.3	914.0	n/a	292.4	3.4
HW/W63	*β*_lower_	0.643	0.771	0.622	1.454	0.594	1.199	0.600
	*β*	0.662	0.804	0.650	1.652	0.629	1.275	0.611
	*β*_upper_	0.682	0.838	0.679	1.866	0.667	1.354	0.622
	*η*_lower_	7,388	11,327	22,576	10,515	51,175	8,277	2,211
	*η* (days)	7,953	12,645	26,253	13,991	65,123	9,405	2,293
	*η*_upper_	8,592	14,207	30,800	19,611	84,455	10,805	2,378
	$B_{50,\mathrm{lower}}$	11.8	20.0	35.9	23.7	80.7	17.3	3.3
	B50 (years)	12.5	22.0	40.9	30.7	99.7	19.3	3.4
	$B_{50,\mathrm{upper}}$	13.4	24.3	46.9	41.8	125.2	21.8	3.6
	MTTSAF_lower_	26.9	34.9	83.5	26.1	195.2	21.1	8.9
	MTTSAF (years)	29.2	39.1	98.3	34.3	252.9	23.9	9.2
	MTTSAF_upper_	31.7	44.1	116.8	47.3	334.5	27.3	9.6
Mechanical	*β*_lower_	0.512	0.690	0.649	1.151	0.554	0.910	0.474
	*β*	0.544	1.011	0.698	1.641	0.608	1.360	0.494
	*β*_upper_	0.578	1.417	0.751	2.253	0.665	1.938	0.513
	*η*_lower_	20,682	43,684	16,604	11,066	43,218	17,734	6,706
	*η* (days)	25,687	188,915	20,797	25,454	62,510	56,200	7,531
	*η*_upper_	32,496	2,022,138	26,696	93,985	94,833	381,088	8,505
	$B_{50,\mathrm{lower}}$	29.9	92.2	27.8	25.7	67.9	40.2	8.9
	B50 (years)	35.9	360.2	33.7	55.8	93.7	117.6	9.8
	$B_{50,\mathrm{upper}}$	43.8	3,269.6	41.8	187.6	134.9	699.8	10.9
	MTTSAF_lower_	95.3	119.3	56.9	27.9	169.7	45.7	36.9
	MTTSAF (years)	121.8	515.3	72.3	62.4	253.3	141.0	42.3
	MTTSAF_upper_	158.9	5,504.0	94.3	219.9	398.0	916.2	48.8
All	*β*_lower_	0.641	0.729	0.626	1.443	0.661	1.021	0.595
	*β*	0.654	0.751	0.643	1.576	0.679	1.073	0.601
	*β*_upper_	0.667	0.774	0.660	1.717	0.698	1.127	0.608
	*η*_lower_	11,035	16,225	17,562	13,109	15,862	16,625	1,962
	*η* (days)	11,675	17,767	19,115	16,281	17,225	19,090	2,008
	*η*_upper_	12,364	19,520	20,882	20,770	18,770	22,139	2,055
	$B_{50,\mathrm{lower}}$	17.4	27.6	27.5	29.0	25.6	32.9	2.9
	B50 (years)	18.3	29.9	29.6	35.3	27.5	37.2	3.0
	$B_{50,\mathrm{upper}}$	19.2	32.4	31.9	44.2	29.6	42.4	3.1
	MTTSAF_lower_	40.8	52.6	66.1	32.5	56.3	44.4	8.0
	MTTSAF (years)	43.4	57.9	72.4	40.0	61.5	50.9	8.3
	MTTSAF_upper_	46.2	63.9	79.8	50.6	67.5	58.9	8.5

### Analysis of fitted distributions

- The distributions reveal HPSS – the most modern POE type – to be the least reliable solution, and mechanical points, the oldest approach, to be the most reliable. The low reliability of HPSS may be due to the observation window coinciding with the roll out of HPSS, and the subsequent final development and testing period with live traffic. A more recent observation window would be required to confirm this.
- The models established in Table 6 can be compared to those independently established by Rama and Andrews.^21^ Notably, the shape parameter β<1 indicates a high infant mortality rate. There are some differences between the *B*_50_ values in the constant and variable failure rate models.
- Comparing the values presented in Table 5 with those in Table 6 indicates that assuming a constant failure rate when modelling switch failures is not an ideal approach, as in all cases the whole-system *β* values are significantly less than 1 – a conclusion which further agrees with those of Rama and Andrews.^21^ This indicates that the accuracy of many predict-and-prevent models used by industry could be significantly improved with the use of variable failure rates.
- Comparing the values presented in Table 5 with those in Table 6 further highlights the weakness of the industry-standard MTTSAF measure – the MTTSAF for mechanical switches at almost 50 years, for instance, would be a misleading value for an asset manager, when considering the *B*_50_ is nearer to 10 years.
- Most elements show a tendency towards β<1, indicating a higher incidence of early life failures. This is not what is expected of an electromechanical device, which would typically be seen to wear out in use. Permanent way elements, with *β* approximately 1, have a broadly constant failure rate.
- An electro-mechanical or electro-hydraulic element showing high infant mortality is an indication of three main possible failure contributors. First, that insufficient burn-in testing is being completed. Second, that there are negative human factors with regard to installation and adjustment, which lead to the components operating outside a design envelope. Third, that the components have not been designed for the correct operating environment. Further analysis would be required to establish which particular cause (or combination thereof) was prevalent.
- ‘Human error’ failures – that is, failures directly attributable to human error rather than those manifesting themselves through the failure of a component – have a relatively high beta. However, the confidence bands of these values are very wide, as there are relatively few failures attributable to this cause. As there is no obvious reason the likelihood of human error should increase with time, it may prove a better approach in future work to fit a constant failure rate model to this element.
- Note that values in the ‘all’ column are calculated using all data points for a given machine to construct a distribution, which because of the mix of *β* values discovered is not an accurate method, a better method being the mixed-Weibull, which is used for the baseline models in the next section.

These subsystem models can now be used to evaluate the benefits of a redundant approach.

## Fault tolerance through redundancy

### Modelling approach

**Figure 7.**
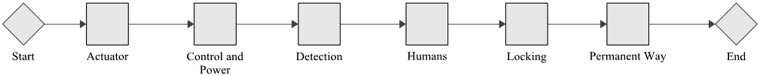
An example RBD showing the baseline case, with a single subsystem of each category. As all subsystems are connected in series, a failure of any one will cause a system failure.

**Figure 8.**
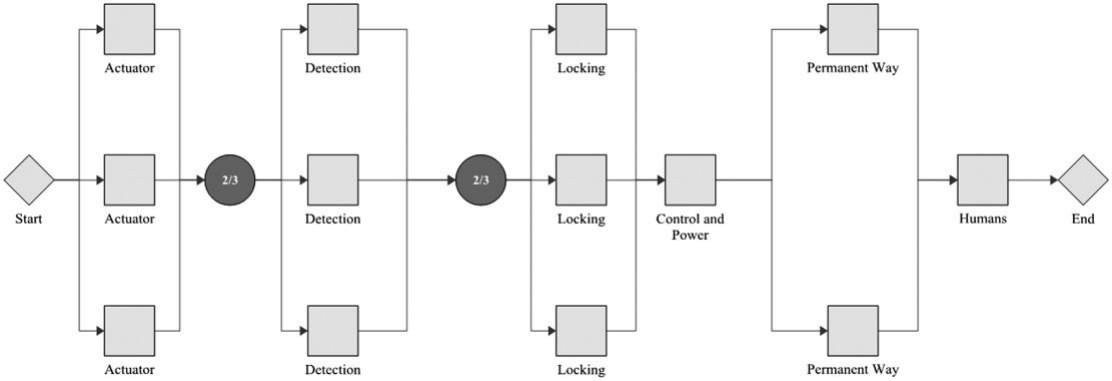
An example RBD showing replication of individual subsystems, with 2-out-of-3 voting (for actuation and detection), triplication (locking) and duplication (permanent way).

With the *β* and *η* values established in the previous section, conceptual designs featuring redundancy of subsystems can now be modelled. This modelling takes an RBD approach. An RBD represents a system by a series of blocks; each block can be in a ‘functional’ or ‘failed’ state. The system is considered to be in a functional state if a path can be created from start (left) to end (right) which encompasses only blocks in the functional state. The modelling considered here is purely analytical, that is it is assumed that no repair of failed subsystems takes place. Three examples of RBDs are provided graphically in this paper; other combinations are represented in shorthand only. This shorthand notation is adopted for brevity, whereby a number (representing number of channels) or fraction (representing *x*-out-of-*y* redundancy) is followed by the abbreviation adopted for each subsystem as used in the source data analysis. Figure 7 shows the baseline example. This has a single instance of each subsystem and would be termed *A C D H L P* in shorthand. As all subsystems are connected in series, a failure of any one will cause a system failure. Another arrangement is shown in Figure 8, which has duplicate, triplicate and 2-out-of-3 elements. The shorthand for this implementation is *2/3A 2/3D 3L C 2P H*.

### Scenarios and strategy

- *Actuation* elements can be combined in parallel-channel redundancy. A range of actuation options can be examined. Singular (i.e. current practice), duplicate, triplicate (including 2-out-of-3) are considered here. However, actuators are also relatively expensive. Cost is not calculated in this paper, but 2-out-of-3 may enable smaller/cheaper units to be utilised.
- *Control/Power* elements could be paralleled in a number of ways; however, it is anticipated that *x*-out-of-*y* approaches would not be suitable due to the complexity of the control signalling. Therefore, the options examined are singular, duplicate and triplicate.
- *Detection* elements can easily be paralleled. However, the sole purpose of detection is to sense the system state, so a problem exists in a duplicate system showing two differing positions, which would likely still be regarded as a failure. Options considered are therefore singular, and the voting systems 2-out-of-3 and 3-out-of-4. The processing element is considered perfect.
- *Human* failures caused by human error must necessarily form part of the system analysis. However, a full analysis of the human factor elements of track switch design, operation, maintenance and repair is not part of this work (see ‘Future Work’ section). The human element is therefore considered consistent with existing practice.
- *Locking* elements can be paralleled. As the fundamental purpose of the lock means a failure could lead to it preventing movement of the switch, it could be deduced that paralleling this subsystem may in fact reduce the overall system reliability. However, in practice, nearly all lock failures result from a lock failing to engage. For this analysis, it is assumed there is an engineering solution to this which enables locks to function as separate units.^7^
- *Permanent Way* elements could be duplicated or triplicated, but no voting approaches could apply as these elements are entirely passive.

**Figure 9.**
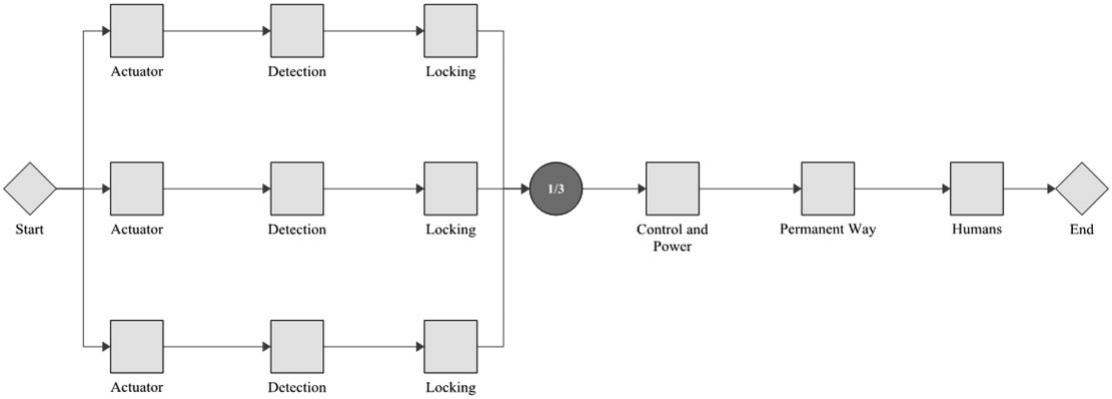
An example RBD showing parallel replication of whole POE units, wherein each unit has actuation, locking and detection elements.

Another approach to be considered (for the power operated points only) is the duplication, triplication and 2-out-of-3 voting for several identical point machines fitted to a single end. This would parallel detection, actuation and locking channels grouped together, in a larger framework of voting and processing, again considered perfect. An example of this approach is shown in Figure 9, the shorthand for which is *3(ADL) C P H*. These grouped elements would each have an associated permanent way, control/power and human elements, which could take the form of the strategies above. It is also not possible to apply each of these strategies to each points type, exceptions are:

- *Actuation* upon the mechanical points type consists of rodding and cable runs from a lever frame to the points. Therefore, a redundancy of actuators would not be practicable.
- *Control/Power* elements upon mechanical points type are rare, yet the failure distribution listed is very low as it is for the whole population analysed. This has therefore been left as a singular item.
- *Locking* elements upon HPSS points type are combined with actuation as established earlier.

When all possible approaches listed above are combined, there are approximately 350 possible permutations per machine type. For brevity, therefore, this paper will present a baseline machine and several concepts for each machine type, demonstrating the most reliable scenarios in each case. Many architectures are evaluated as each will incur a different monetary cost; evaluating relative cost is the subject of further work. The scenarios were selected by way of a sensitivity analysis of each subsystem, which iteratively examined the static contribution to unreliability of each subsystem. The process for creating the distributions is based on the Monte-Carlo approach. The completed RBDs are used, with random inputs, to predict first failure times of the system. This process is repeated until a dataset of 500 simulated failure points is created, for each combination. This dataset can then be subject to the same MLE process detailed earlier, in order to calculate the *β* and *η* parameters, and *B*_50_ values. For completeness, *MTTSAF* figures are also calculated. Note that this is a different method to that used to calculate the ‘All’ column in Table 4, which was to fit a single 2P-Weibull distribution to a dataset which was known to be a mix of different distributions. The results of the two processes are therefore expected to be marginally different. Table 4 can be used to validate the Monte-Carlo approach.

### Static versus dynamic analysis

One of the benefits of a multi-channel approach is that the system continues to function until such a time as a repair has been effected, unless all channels fail simultaneously. Whilst a static analysis can reveal the expected system reliability, a more relevant measure can be obtained from a dynamic simulation – using the same RBD and Monte-Carlo approach – to establish the availability. To establish availability, the benchmark MTTR is used as the time to fix any failed subcomponent. As the failure distributions are significantly time-variant, the dynamic simulations are run over an observation window of 25 years (a typical asset lifetime for a switch installation) and the mean unavailability per annum, in minutes, taken as a measure for comparison. Note that this availability figure relates to unscheduled downtime only and does not allow for scheduled maintenance downtime, which is considered part of the system.

## Analysis

**Figure 10.**
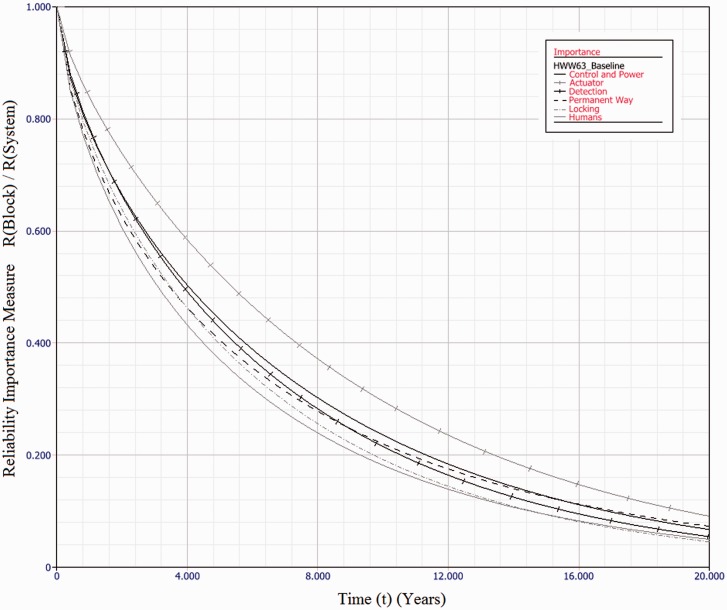
Reliability Importance of each subsystem type for baseline case of HW/W63 machine type, over 20 years of operation. As it is a series system, all elements contribute similar levels of unreliability at each point in time.

**Figure 11.**
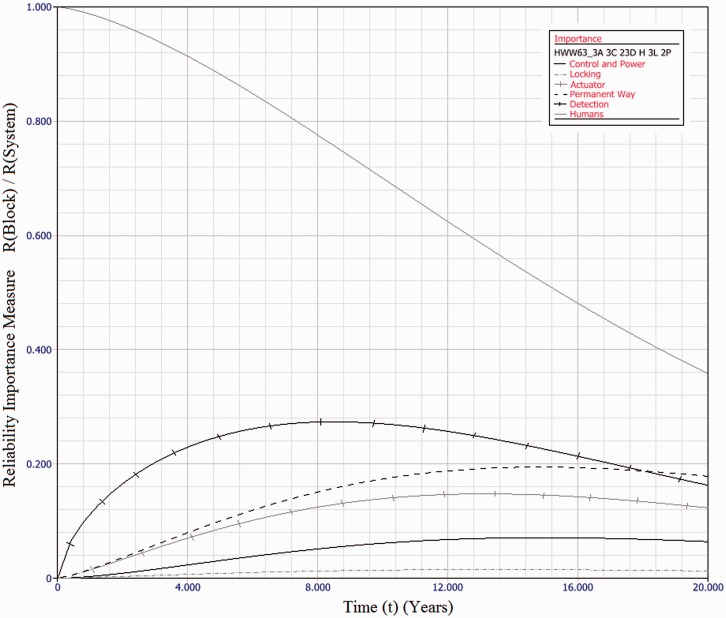
Reliability Importance of each subsystem type for *3A 3C 2/3D H 3L 2P* case of HW/W63 machine type, over 20 years of operation. System reliability is dominated by human error over the entire time period.

The results of the static modelling are presented in Table 7. The results of the dynamic modelling are presented in the right-hand column of Table 7. The *B*_50_ figures show that redundancy can provide a considerable improvement over baseline for every POE type. The mean annual downtime for each redundantly engineered solution is an order of magnitude lower than the baseline scenario. The following further points are of note:

- Industry practice is to use mean-times as a measure of reliability. However, with highly skewed distributions, as calculated here, this measure can be significantly misleading. This paper suggests use of the *B*_50_ value as a more representative measure, whether or not failure rates are considered constant.
- In all cases, parallel redundancy of functional subsystems acts to improve overall system reliability.
- For HPSS, fitting three machines in a parallel configuration results in a fivefold improvement in *B*_50_ value.
- For the HW/W63 electromechanical machines, up to 12.5 year *B*_50_ values are achievable, a fivefold improvement.
- For Clamplock/Hydrive types, *B*_50_ can also exceed 10 years, also a fivefold improvement.
- As expected, different architectures have different effects upon whole system reliability. To select a suitable architecture for a given situation, cost constraints must also be taken into account, alongside the maintenance and repair policy.
- Figure 10 shows the relative reliability importance of each subsystem type, for the HW/W63 baseline example. Reliability importance is calculated as the subsystem reliability divided by system reliability and gives an indication of how likely a failure of that subsystem is to cause a system failure. It can be seen that for the series case, the failure of any block is of similar likelihood to cause a system failure at any point in the observation window. This result is to be expected for a series system.
- Figure 11 shows the relative reliability importance for a sample case, *3A 3C 2/3D H 3L 2P* of the HW/W63 POE type. The importance of all physical subsystems has been considerably reduced, indicating a good fault tolerance. However, the human element is now of dominant importance throughout the observation window. The same is true for *all* evaluated architectures, as it is not possible to add redundancy to the human element in the same way. There is also the possibility that the Human element would be less reliable with a multi-channel system, as the extra complexity may lead to additional human error. Adding additional redundancy beyond that explored herein does not significantly further improve system reliability, as the Human element becomes the limiting factor. This result is important in indicating that when implementing functionally redundant track switching solutions, human factors elements are important in gaining the full reliability benefits. Any neglect of human factors in this instance may mean that there may be no reliability improvement at all.
- The results of the dynamic modelling show that an order of magnitude reduction in unscheduled downtime is possible across all asset types, when a functionally redundant design approach is taken.
- The dynamic modelling also shows that the particular architecture has a relatively insignificant effect upon the unscheduled downtime for each switch type. This is because the likelihood of parallel channels failing concurrently, within the comparatively short MTTR, is diminishingly small.
- The main contributor to the unscheduled downtime in each scenario is errors directly attributable to humans. This is further highlighted in Figure 11. HPSS performs better than the other drive types in the mean unavailability per annum due to the fact the eta value for human-induced failures is much higher – there is less likelihood of error as the machine has built-in monitoring and diagnostics.
- As the MTTR is insignificantly small when compared with the MTTSAF, there may be some scope in a multi-channel architecture to respond to subsystem failures in a much longer time frame – perhaps weeks or months – without having a significant detrimental effect upon availability. Further modelling work will be necessary to establish this relationship.
- This modelling has not considered the practical limitations to implementation. Of note is the fact that providing redundancy in locking with existing designs may not be possible. A novel design of locking system allowing multiple channels would therefore be required. The proposed ‘REPOINT’ design, first presented in Bemment et al.,^7^ is one option which enables a redundancy of locking systems.

**Table 7.** *β, η*, MTTSAF and B50 values for a selection of redundantly engineered switch solutions based upon existing POE types.

Machine type	Concept structure	Static				Dynamic
		*β*	*η*	MTTSAF	B50	Mean Unavailibility
			(days)	(years)	(years)	(min per annum)
Clamplock/Hydrive	A C D H L P (Baseline)	0.750	1,136	3.7	1.9	24.1
	2/3A 3C 2/3D H 2L 2P	1.253	2,587	6.7	5.1	1.3
	2/3A 3C 2/3D H 3L 2P	1.276	2,861	7.4	5.7	1.3
	2/3A 3C 3/4D H 2L 2P	1.252	2,345	6.1	4.7	1.3
	2/3A 3C 3/4D H 3L 2P	1.270	2,572	6.6	5.2	1.3
	3A 3C 2/3D H 2L 2P	1.353	4,181	10.6	8.6	1.3
	3A 3C 2/3D H 3L 2P	1.468	4,802	12.1	10.0	1.3
	3A 3C 3/4D H 2L 2P	1.319	3,603	9.2	7.3	1.3
	3A 3C 3/4D H 3L 2P	1.393	4,088	10.3	8.5	1.3
	2/3(ADL) 3C H 2P	1.152	1,319	3.6	2.5	1.3
	3(ADL) 3C H 2P	1.431	3,610	7.3	9.3	1.3
HPSS	A C D H P (Baseline)	0.623	555	2.1	0.9	36.2
	2/3A 3C 2/3D H 2P	1.055	1,104	2.1	3.1	0.9
	2/3A 3C 3/4D H 2P	1.037	710	2.0	1.3	0.9
	3A 3C 2/3D H 2P	1.033	1,261	3.5	2.3	0.8
	3A 3C 3/4D H 3P	1.020	770	2.2	1.4	0.9
	2/3(AD) 3C H 2P	1.026	863	2.5	1.6	0.9
	3(AD) 3C H 2P	1.256	2,531	6.7	4.9	0.9
HW/W63	A C D H L P (Baseline)	0.716	1,568	5.0	2.7	18.9
	2/3A 3C 2/3D H 2L 2P	1.036	3,618	9.5	7.1	1.8
	2/3A 3C 2/3D H 3L 2P	1.035	3,682	9.7	7.3	1.8
	2/3A 3C 3/4D H 2L 2P	1.037	3,251	8.6	6.4	1.9
	2/3A 3C 3/4D H 3L 2P	1.036	3,303	8.8	6.5	1.9
	3A 3C 2/3D H 2L 2P	1.114	5,845	14.7	12.2	1.7
	3A 3C 2/3D H 3L 2P	1.119	5,995	15.0	12.5	1.7
	3A 3C 3/4D H 2L 3P	1.086	5,025	12.8	10.3	1.9
	3A 3C 3/4D H 3L 2P	1.078	5,143	13.1	10.6	1.8
	2/3(ADL) 3C H 2P	0.989	2,398	6.6	4.5	1.9
	3(ADL) 3C H 2P	1.155	5,603	14.1	11.6	1.8
Mechanical	A C D H L P (Baseline)	0.621	3,357	12.5	5.3	8.1
	A C 2/3D H 2L 2P	0.685	5,193	16.6	9.0	3.7
	A C 2/3D H 3L 2P	0.680	5,412	17.3	9.4	3.6
	A C 3/4D H 2L 2P	0.716	4,148	12.8	7.3	3.9
	A C 3/4D H 3L 2P	0.713	4,282	13.3	7.5	3.7
	A 2/3(DL) C H 2P	0.713	3,967	12.5	6.9	3.8
	A 3(DL) 3C H 2P	0.648	7,090	23.1	12.4	3.8

*MTTSAF*: mean time to service affecting failure, describes how often the system can be expected to suffer a failure which is service affecting (operational reliability).

## Conclusions

This paper has established that adopting a fault tolerant approach to railway track switching is able to bring considerable gains in reliability and availability. Reliability of track switches is a problem on the UK mainline, causing much delay to trains and with an associated cost to the infrastructure manager. This paper has analysed failure data from the UK mainline infrastructure custodian, covering 74,800 years of operation, in order to establish failure distribution parameters and reliability figures for different switch machine types when decomposed into their functional subsystems. These parameters have then been used as inputs to a range of RBD models which establish analytically the increase to reliability possible when taking a parallel-subsystem approach to fault tolerance. The results show that considerable gains in whole-system reliability are demonstrated in a range of possible implementations; typical time to failures can be more than five times that of existing solutions, and unscheduled downtime reduced by an order of magnitude. However, as equipment failures are engineered out, switch reliability can be seen to plateau. This is due to the dominant contributor to unreliability becoming human error, which cannot be designed out in the same manner. As considerable reliability gains are demonstrated, this paper makes a strong case for developing track switch designs utilising functional redundancy. The potential impact of such designs on reliability and availability is significant.

## Future work

Future work investigating fault tolerant track switching will centre around three main areas:

- Dynamic reliability modelling of suggested architectures for fault tolerant track switching solutions. The work contained herein is analytical only, and clearly one of the main benefits to the implementation of parallel-channel redundancy is the extension of the window where repair/replacement can occur. This work will require extensive modelling but build directly upon the failure distributions established in this paper.
- A more detailed engineering appraisal of the physical constraints of fault tolerant track switching needs to be carried out. This paper does not consider for example the space, cost or time constraints within which the track switching solutions must perform, or indeed whether engineering a physical embodiment of the proposed redundant architectures is possible.
- Seek a greater understanding of the human factors elements of track switch installation, maintenance and repair. In any future implementation, minimising the human contribution to failures will be just as important as engineering out service affecting failures, as demonstrated by this paper.
